# Human Myeloma Cell Lines Induce Osteoblast Downregulation of CD99 Which Is Involved in Osteoblast Formation and Activity

**DOI:** 10.1155/2015/156787

**Published:** 2015-04-27

**Authors:** Angela Oranger, Giacomina Brunetti, Claudia Carbone, Graziana Colaianni, Teresa Mongelli, Isabella Gigante, Roberto Tamma, Giorgio Mori, Adriana Di Benedetto, Marika Sciandra, Selena Ventura, Katia Scotlandi, Silvia Colucci, Maria Grano

**Affiliations:** ^1^Department of Basic Medical Sciences, Neurosciences and Sense Organs, Section of Human Anatomy and Histology, University of Bari, Piazza Giulio Cesare 11, 70124 Bari, Italy; ^2^Department of Clinical and Experimental Medicine, Medical School, University of Foggia, Viale Luigi Pinto 1, 71100 Foggia, Italy; ^3^CRS Development of Biomolecular Therapies, Laboratory of Experimental Oncology, Orthopaedic Institute of Rizzoli, Via di Barbiano 1/10, 40136 Bologna, Italy

## Abstract

CD99 is a transmembrane glycoprotein expressed in physiological conditions by cells of different tissues, including osteoblasts (OBs). High or low CD99 levels have been detected in various pathological conditions, and the supernatant of some carcinoma cell lines can modulate CD99 expression in OB-like cells. In the present work we demonstrate for the first time that two different human myeloma cell lines (H929 and U266) and, in a less degree, their conditioned media significantly downregulate CD99 expression in normal human OBs during the differentiation process. In the same experimental conditions the OBs display a less differentiated phenotype as demonstrated by the decreased expression of RUNX2 and Collagen I. On the contrary, when CD99 was activated by using a specific agonist antibody, the OBs become more active as demonstrated by the upregulation of Alkaline Phosphatase, Collagen I, RUNX2, and JUND expression. Furthermore, we demonstrate that the activation of CD99 is able to induce the phosphorylation of ERK 1/2 and AKT intracellular signal transduction molecules in the OBs.

## 1. Introduction

CD99 is a 32 kDa transmembrane glycoprotein, encoded by the MIC2 gene [1] which is located on the pseudoautosomal regions of both human X and Y chromosomes [2]. MIC2 gene encodes two distinct proteins produced by alternative splicing of the CD99 gene transcript [3] and, compared with the major wild-type full-length form, the minor splice variant form of CD99 has a relatively short intracytoplasmic fragment [4].

It is well known from the literature that CD99 can be expressed in both normal and pathological tissues. In normal tissues it is expressed in cortical thymocytes, pancreatic islet cells, granulose cells of ovary, Sertoli cells of testis, CD34^+^ cells of bone marrow, and all leukocyte lineages [5].

Slightly less than a decade ago its linkage to human osteoblast (hOB) differentiation has emerged and MIC2 has been indicated under the control of the transcription factor RUNX2, which is essential for hOB differentiation [6, 7].* In vitro* data have demonstrated CD99 expression in cell-adhesion structures of osteoblastic cell cultures, and* in vivo* its expression has been highly detected in hOBs adhering to each other and lining the bone surface in tissue samples [5]. Very recently, we have shown that during the differentiation process hOBs and bone marrow-mononuclear cells increased CD99 expression levels, suggesting its role in osteoblastogenesis [8]. Accordingly, CD99 expression is high in lining cells and in mature hOBs [5, 8].

Quite variable CD99 levels have been demonstrated in pathological conditions since they have been detected either high or low. In particular, it is overexpressed in different sarcomas, such as Ewing's sarcoma [9, 10], synovial sarcoma [11], mesenchymal chondrosarcoma [12], and rhabdomyosarcoma [13], in lymphoblastic lymphoma/leukemia [14], and human breast cancer cells [15]. Conversely, CD99 is downregulated or completely lacking in pancreatic endocrine neoplasm [16], gastric adenocarcinoma [17], and osteosarcoma [5].

Literature evidences have also demonstrated that the supernatants from several carcinoma cell lines associated with osteolytic metastases (breast, colon, pancreatic, renal, and hepatoma cell lines) specifically downregulate CD99 on AHTO-7 cells (large T antigen transfected human trabecular OBs) [18]. Conversely, the conditioned media obtained from prostate cancer cell lines, which correlated with osteosclerotic lesions, induce an increase in CD99 expression [18]. A very recent paper performed on a large number of MM patients suggests CD99 as a new marker for risk stratification of disease severity [19]. However, no data are at present available on the effect of myeloma cells on OB CD99 expression and the possible implication of CD99 in the impairment of OB differentiation in multiple myeloma (MM), a hematological B cell malignancy associated with bone disease [20–27].

It is well established that myeloma cells, through a variety of cellular mechanisms, contribute to the onset of osteolytic bone lesions by altering bone remodeling as they induce both the increase of osteoclastic bone resorption and decrease of OB differentiation. With particular regard to the alteration of osteoblastic differentiation and function in MM bone disease it has been showed that, among the numerous cytokines, myeloma cells produce soluble molecules such as frizzled-related proteins-2 and -3 (sFRP-2 and -3) [27–29], Dickkopf-1 (DKK-1) [28], and sclerostin [25–27], responsible for the inhibition of the canonical Wingless-type (Wnt) signaling which is a crucial pathway for the correct OB differentiation and activity.

Not later than a few months ago, it was found that, under physiological conditions, OBs express increasing levels of CD99 during their differentiation [8] but regarding the influence of myeloma cells on the expression of this molecule currently lack experimental evidence. Therefore, having previously performed studies on the mechanisms altering OB differentiation contributing to osteolytic process in MM bone disease [25, 26], we here focused our interest on this issue. However, it should be considered into account that most information about CD99 activities derives from triggering CD99-mediated signaling events with agonistic CD99 monoclonal antibodies. By using these molecules it has been shown that CD99 is functionally implicated in the apoptosis of cells with immunological role (thymocytes, T-lymphocytes, Jurkat cell line, and normal and leukemia B cell precursors) [30–33], in triggering homotypic CD4+ CD8+ thymocytes aggregation [34], in inducing T cell migration to inflamed vascular endothelium [35], and in cell-to-cell contact and diapedesis of monocytes across endothelial cells by homophilic interactions between these two adjacent cells [36]. In addition to these effects, several evidences have also proved the role of CD99 in proliferation and activation of lymphocytes [37, 38] and regulation of MHC class I molecule transport from the Golgi complex to the cell surface [39].

Thus, on the basis of all these findings and the intriguing role of CD99 in osteogenesis and bone pathophysiology, in this paper we analyzed the influence of human myeloma cell lines (HMCLs) on CD99 expression by hOBs. We demonstrated that HMCLs display the ability to reduce the expression of CD99 in normal hOBs during the differentiation process. Furthermore, by using an anti-CD99 agonist monoclonal antibody, we demonstrated that the hOBs result more active in the expression of their differentiation parameters. These data suggest that CD99 can be important in the differentiation and activity of hOBs in physiological and pathological conditions.

## 2. Materials and Methods

### 2.1. Human Osteoblasts

Trabecular bone specimens, obtained from healthy subjects who undergo femur surgery following traumatological events, were cleaned off soft tissues, reduced to small fragments, and digested with 0.5 mg/mL* Clostridium histolyticum* neutral collagenase (Sigma Chemical Co., St. Louis, MO, USA) in minimum essential medium (*α*-MEM) (Gibco Invitrogen, Milan, Italy) with gentle agitation for 30 minutes at 37°C. Bone fragments were then washed (three times) with phosphate-buffered saline (PBS) and cultured in *α*-MEM supplemented with 10% fetal calf serum (FCS) (Gibco), 100 IU/mL penicillin (Gibco), 100 mg/mL streptomycin (Gibco), and 2.5 mg/mL amphotericin B (Gibco), at 37°C in a water-saturated atmosphere containing 5% CO_2_. Cells were fed by medium replacement every 3 to 4 days. In these conditions, the hOBs resident in the explants proliferated and migrated to the culture substrate, reaching confluence within 3 to 4 weeks. Cells were then trypsinized and transferred to appropriate culture dishes for characterization and experiments.

Informed consent to the study was given according to the tenets of the Declaration of Helsinki. Approval was obtained from the Institutional Review Board of the Laboratory of Experimental Oncology, Rizzoli Orthopaedic Institute, Bologna, Italy (Protocol number 0021571 of June 28, 2013).

### 2.2. Cell Culture Conditions and Cocultures

H929 and U266 HMCLs were cultured in RPMI 1640 medium supplemented with 10% FCS and then lysed for protein extraction or used for coculture experiments.

Confluent hOBs were cultured in the presence or absence of 5 × 10^3^/cm^2^ HMCLs (H929 or U266) or their conditioned medium, in osteogenic medium consisting of *α*-MEM medium supplemented with 10% FCS, 50 *μ*g/mL ascorbic acid, and 10^−8^ M dexamethasone (all from Sigma), for 2, 7, 14, and 21 days before lysing them for protein extraction. In parallel, other hOBs were cultured in *α*-MEM medium supplemented with 10% FCS and lysed for protein extraction after their adhesion (0 days of differentiation).

Moreover hOBs were cultured at a density of 1 × 10^4^ cells/cm^2^ in 96- or 48-well plates in *α*-MEM medium supplemented with 10% FCS, in the presence or absence of 2 *μ*g/mL anti-CD99 (DN-16) (Abcam, Cambridge Science Park) agonist monoclonal antibody or mouse IgG (Sigma) and after 24 and 48 hours or after 6 days of culture were analyzed, respectively, for 3-(4,5-dimethylthiazol-2-yl)-2,5-diphenyltetrazolium bromide (MTT) assay or Alkaline Phosphatase- (ALP-) staining. These cells were also plated in 6 well plates and after reaching 70% of confluence were treated with 2 *μ*g/mL of anti-CD99 agonist monoclonal antibody or mouse IgG or 100 ng/mL human TNF-related apoptosis-inducing ligand (h-TRAIL), for 4, 6, 12, and 24 hours and then lysed for protein extraction to evaluate apoptotic pathway. In addition, the stimulation with CD99 agonist antibody or mouse IgG was also performed for 2, 5, 10, and 20 minutes and these cells were then lysed for protein extraction to study AKT and ERK phosphorylation. hOBs were also treated with 100 nM Wortmannin (Calbiochem, Germany) for 30 minutes or with 10 *μ*M PD98059 (Sigma) for 60 minutes, respectively, Phosphoinositide 3-Kinase (PI3K) and extracellular signal-regulated kinase-1 (ERK1) K inhibitors, and then stimulated with 2 *μ*g/mL anti-CD99 agonist monoclonal antibody for 4 and 6 hours. Before the short times (2, 5, 10, and 20 minutes) stimulation, cells were starved for 12 hours with *α*-MEM medium supplemented with 2% FCS and thus treated as previously described using the same medium.

All stimulation experiments were repeated for three times.

### 2.3. Western Blot Analysis

The protein levels of CD99 have been evaluated in H929 and U266. Additionally, CD99, Collagen I (COLLI), and RUNX2 protein levels have been also analyzed in hOBs cultured alone and cocultured with HMCLs or their conditioned medium. All the cells were solubilized with lysis buffer [50 mM Tris (tris(hydroxymethyl)aminomethane)-HCl (pH 8), 150 mM NaCl, 5 mM ethylenediaminetetraacetic acid, 1% NP40, and 1 mM phenylmethyl sulfonyl fluoride]. Moreover, COLLI, RUNX2, members of AP1 complex (FRA1, FRA2, and JUND), and mitogen-activated protein kinases (MAPKs) have been studied in hOBs after CD99 stimulation. COLLI, RUNX2, and JUND were also evaluated in hOBs treated for 30 minutes with 100 nM Wortmannin or for 60 minutes with 10 *μ*M PD98059 and then stimulated for 4 and 6 hours with anti-CD99 agonist monoclonal antibody; these cells were solubilised with the lysis buffer previously described.

To detect the expression of caspases 3, 7, and 8 and Bid cleavage in hOBs after CD99 or hTRAIL stimulation, cells were lysed by incubation on ice for 30 min in lysis buffer containing 20 mM Tris-HCl (pH7.5), 1% Triton X-100, 150 mM NaCl, 10% glycerol, 1 mM Na_3_VO_4_, 50 mM NaF, 100 mM phenylmethylsulfonyl fluoride, and a commercial protease inhibitor mixture.

Cell proteins (15 *μ*g) were subjected to sodium dodecyl sulfate-polyacrylamide gel electrophoresis (SDS-PAGE) gel and subsequently transferred to nitrocellulose membranes (Hybond; Amersham Pharmacia, London, UK). The blots were probed overnight at 4°C with the appropriate primary antibody.

The following primary antibodies were used: monoclonal anti-COLLI, anti-p-ERK and anti-*β*-Actin, polyclonal anti-JUND, anti-FRA1, anti-FRA2, and anti-total-ERK (all from Santa Cruz Biotechnology, Santa Cruz, CA, USA); monoclonal anti-p-AKT, anti-p-JNK, and anti-caspase-8, polyclonal anti-total-AKT, anti-p-P38, anti-caspase-3, anti-caspase-7, and anti-Bid (all from Cell Signaling, San Diego, CA, USA); monoclonal anti-CD99 (12E7) (Santa Cruz Biotechnology); and polyclonal anti-RUNX2 (Abnova, Taiwan).

After incubation with the appropriate fluorescent-dye-conjugated secondary antibody (LI-COR Biosciences GmbH, Bad Homburg, Germany), specific reactions were revealed with the LI-COR's Odyssey Infrared Imaging System (LI-COR Biotechnology, Lincoln, NE, USA).

### 2.4. Alkaline Phosphatase

ALP was histochemically assessed in hOBs treated and nontreated for 6 days with anti-CD99 agonist antibody or with mouse IgG, using Leukocyte Alkaline Phosphatase Kit, a commercial kit based on naphthol AS-BI and fast red violet LB (Sigma).

Cells were fixed with a citrate-acetone-formaldehyde fixative for 30′′ at room temperature. After being gently rinsed with distilled water, cells were incubated for 15′ in dark with alkaline-dye mixture (NaNO_2_, FRV-Alkaline Solution, Naphthol AS-BI Alkaline Solution) and finally washed with water.

The quantification and normalization of ALP histochemical staining was done counting ALP positive cells respect to total cells in three different fields (10x). Three different experiments were performed for ALP evaluation.

### 2.5. Cell Viability Assay

Mitochondrial dehydrogenases activity was determined by MTT assay. This assay is based on the ability of forming dye crystals to be developed only in living cells, providing an indication of the mitochondrial integrity and activity which, in turn, may be interpreted as a measure of cell viability. hOBs were cultured in 96-well tissue-culture plates as previously described. A part of the wells were used as control, while the others were treated with 2 *μ*g/mL of anti-CD99 agonist antibody for 24 and 48 hours. The cell viability experiments were performed in the presence of 10% FCS. MTT 0.5 mg/mL were added to the culture media followed by 4 hours incubation at 37°C in a humidified 5% CO_2_ atmosphere. The reaction was stopped by the addition of 150 *μ*L of 0.04 N HCl in absolute isopropanol. The optical density was read at 570 nm using an automatic plate reader (550 Microplate Reader Bio-Rad Laboratories Inc., CA, USA). The results were compared to cells incubated under control conditions.

Cell viability was evaluated in three independent experiments.

### 2.6. Statistical Analyses

Statistical analyses were performed by Student's *t*-test with the Statistical Package for the Social Sciences (spssx/pc) software (SPSS, Chicago, IL, USA). The results were considered statistically significant for *P* < 0.05.

## 3. Results

### 3.1. Effect of Human Myeloma Cell Lines on CD99 Expression during Osteoblast Differentiation

It was shown that some carcinoma cell line-conditioned media downregulate CD99 on human AHTO-7 OBs [18]. Thus, after demonstrating that both H929 and U266 express very low protein levels of CD99 (data not shown), we studied the influence of HMCLs on CD99 expression by normal hOBs during their differentiation process.

**Figure 1:**
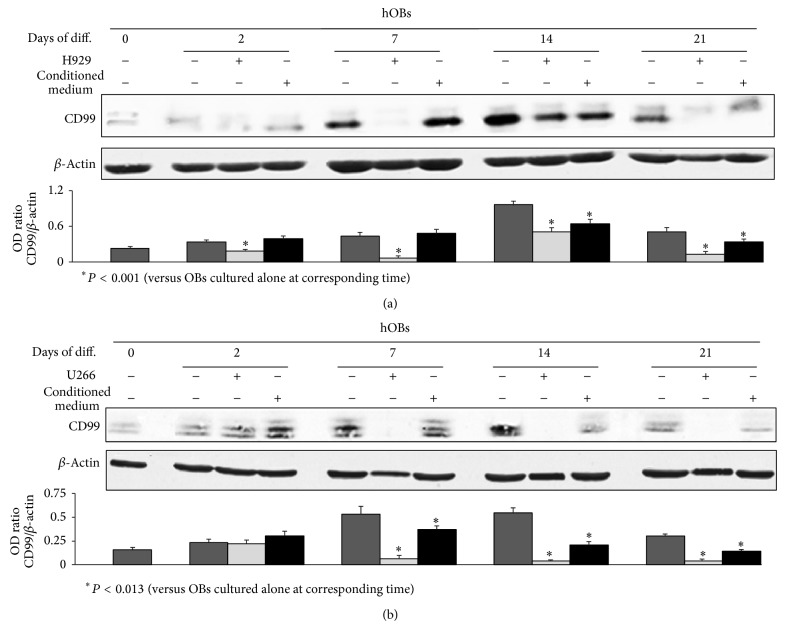
Human myeloma cell lines (HMCLs) inhibit CD99 expression on normal human osteoblasts (hOBs). Human undifferentiated osteoblasts or hOBs differentiated for 2, 7, 14, or 21 days (Days of diff.), were cultured in the presence or absence of H929 (a) or U266 (b) HMCLs or their conditioned medium, and then were analyzed for western blot analysis to detect the protein levels of CD99. The histograms represent the mean optical density (OD) of CD99 ratio normalized to the OD of *β*-Actin. Data are presented as mean ± SE. The figure shows one representative of three independent experiments.

At this purpose we performed cocultures between two different HMCLs (H929 and U266) and undifferentiated normal hOBs cultured in the presence of osteogenic medium from 2 up to 21 days of culture. By western blot analysis we showed that in the coculture system both HMCLs significantly (*P* < 0.013) inhibited hOB CD99 expression during the differentiation period reaching the maximum inhibition at the seventh day of culture (Figure 1). Although the inhibition induced by the two HMCLs was quite similar, the effect exerted by U266 was more pronounced and persistent throughout the whole differentiation period (Figure 1). In parallel, in the same experiment we cultured the hOBs in the presence of the conditioned media collected from the previously mentioned HMCLs to understand if the effect could be mediated by soluble factors. The conditioned medium of both HMCLs displayed a weaker but significant (*P* < 0.013) inhibition of CD99 expression in the late phase of hOB differentiation (14 and 21 days) compared to what was observed in the presence of the cells (Figure 1). These findings suggest that the inhibition on the CD99 expression by hOBs exerted by HMCLs could be partially mediated by soluble factors and further enhanced by the presence of malignant cell lines.

**Figure 2:**
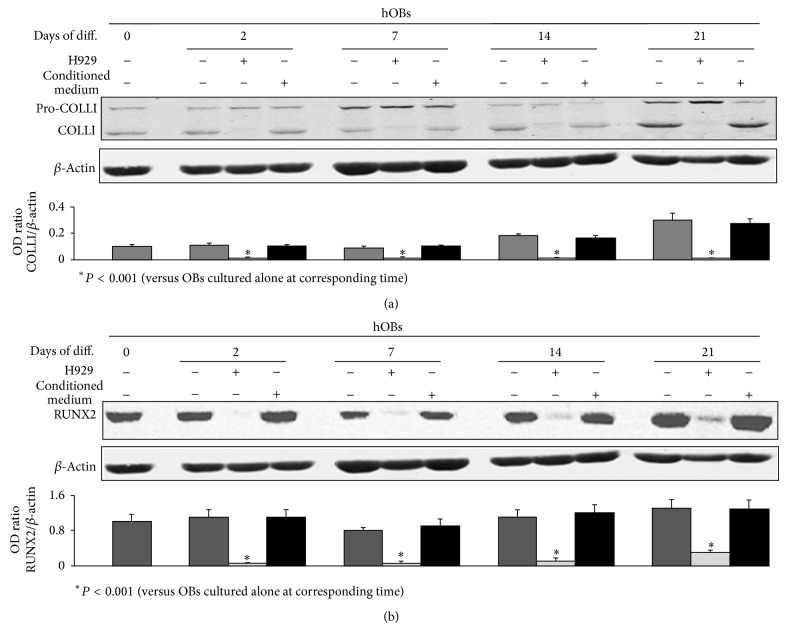
H929 inhibit Collagen I (COLLI) and RUNX2 expression on normal human osteoblasts (hOBs). Human undifferentiated osteoblasts or hOBs differentiated for 2, 7, 14, or 21 days (Days of diff.), were cultured in the presence or absence of H929 or their conditioned medium, and then were analyzed for western blot analysis to detect the protein levels of COLLI (a) or RUNX2 (b). The histograms represent the mean optical density (OD) of COLLI or RUNX2 ratio normalized to the OD of *β*-Actin. Data are presented as mean ± SE. The figure shows one representative of three independent experiments.

**Figure 3:**
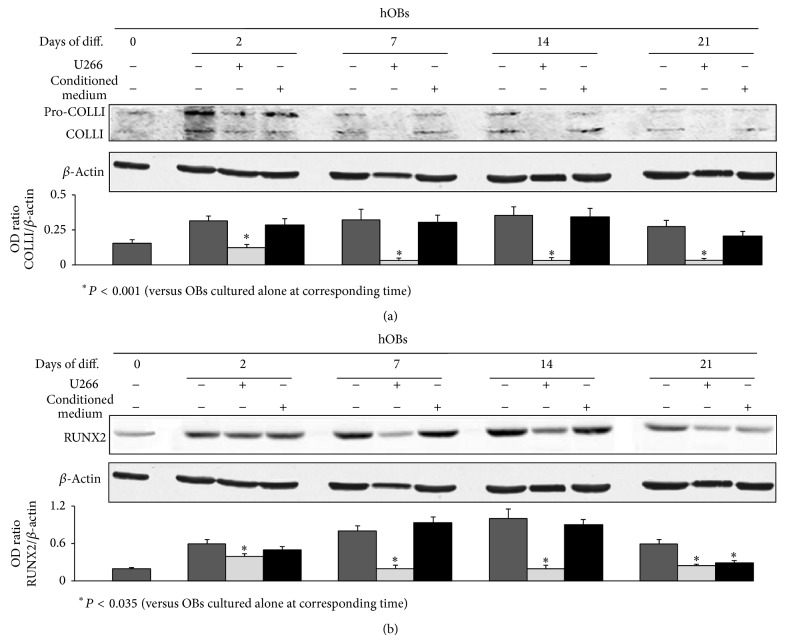
U266 inhibit Collagen I (COLLI) and RUNX2 expression on normal human osteoblasts (hOBs). Human undifferentiated osteoblasts or hOBs differentiated for 2, 7, 14, or 21 days (Days of diff.), were cultured in the presence or absence of U266 or their conditioned medium, and then were analyzed for western blot analysis to detect the protein levels of COLLI (a) or RUNX2 (b). The histograms represent the mean optical density (OD) of COLLI or RUNX2 ratio normalized to the OD of *β*-Actin. Data are presented as mean ± SE. The figure shows one representative of three independent experiments.

Using the previously described coculture system, we also evaluated the effect of HMCLs or their conditioned medium on the expression of COLLI and RUNX2 in hOBs during their differentiation period. By western blot analysis we showed that both H929 (Figure 2) and U266 (Figure 3) significantly inhibited COLLI (*P* < 0.001) (Figures 2(a) and 3(a)) and RUNX2 (*P* < 0.035) (Figures 2(b) and 3(b)) protein levels during the entire differentiation period, whereas the conditioned medium of both HMCLs does not display any effect (Figures 2 and 3). Any toxic-or apoptotic-induced effect of HMCLs on OB cultures was excluded by MTT assay (data not shown).

### 3.2. Effect of CD99 Activation on Normal Human Osteoblasts

**Figure 4:**
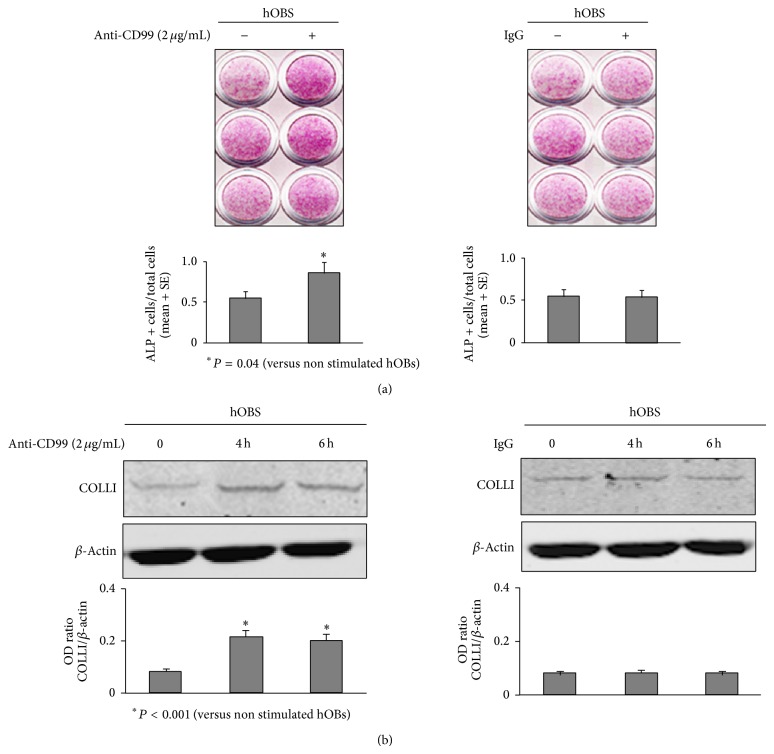
CD99 agonist monoclonal antibody increases Alkaline Phosphatase (ALP) activity and Collagen I (COLLI) expression in normal human osteoblasts (hOBs). (a) Histochemical staining for ALP in differentiated normal hOBs treated for 6 days with or without anti-CD99 agonist monoclonal antibody or mouse IgG. This experiment has been performed in triplicate. The histograms represent the number of ALP positive cells respect to total cells ± SE in three different fields (10x), of three independent experiments. (b) hOBs, treated with anti-CD99 agonist monoclonal antibody or with mouse IgG for 0, 4, and 6 hours (h), were lysed and analyzed by western blot analysis to detect the protein levels of COLLI. The histograms represent the mean optical density (OD) of COLLI ratio normalized to the OD of *β*-Actin. Data are presented as mean ± SE. The figure shows one of three independent experiments.

We have recently demonstrated that CD99 expression increases during normal hOB differentiation [8]; thus we here evaluated whether the activation of CD99 can have an impact on the activity of differentiated normal hOBs. In particular, we treated these cells with anti-CD99 agonist antibody to analyze ALP activity and COLLI expression compared to hOBs cultured in the absence of the agonist antibody as control condition. By using a histochemical assay, we demonstrated significantly (*P* = 0.04) higher ALP activity in hOBs treated for 6 days with 2 *μ*g/mL of anti-CD99 agonist antibody compared to the control. In parallel, to exclude any nonspecific effect of the antibody, the cells were cultured for 6 days with mouse IgG and we did not find any difference (Figure 4(a)).

We have also evaluated the protein expression levels of COLLI, which is the most abundant protein produced by the hOBs [40, 41]. At this purpose, by western blot analyses we demonstrated that, in hOBs treated for 4 and 6 hours with 2 *μ*g/mL of anti-CD99 agonist antibody, COLLI expression was significantly (*P* < 0.001) upregulated respect to untreated cells, and the treatment with the mouse IgG did not induce any effect (Figure 4(b)).

**Figure 5:**
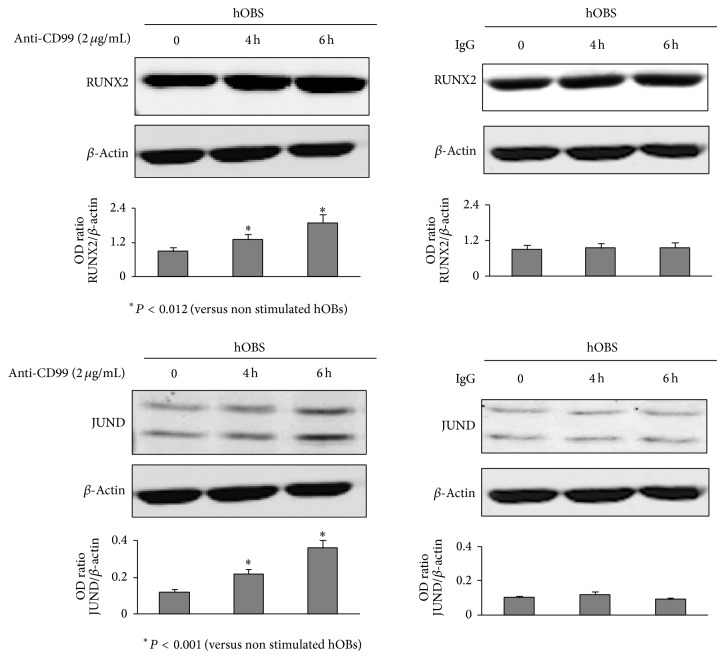
CD99 agonist monoclonal antibody increases RUNX2 and JUND expression in normal human osteoblasts (hOBs). hOBs, treated with anti-CD99 agonist monoclonal antibody or with mouse IgG for 0, 4 and 6 hours (h), were lysed and analyzed by western blot analysis to detect the protein levels of RUNX2 (upper panel) and JUND (lower panel) OB trascription factors. The histograms represent the mean optical density (OD) of RUNX2 or JUND ratio normalized to the OD of *β*-Actin. Data are presented as mean ± SE. The figure shows one representative of three independent experiments.

On the basis of these results, we evaluated whether the activation of CD99 could also affect the expression of transcription factors regulating hOB differentiation, such as RUNX2, and members of AP1 complex such as JUND, FRA1, and FRA2. hOBs treated for 4 and 6 hours with 2 *μ*g/mL of anti-CD99 agonist antibody displayed significantly higher RUNX2 and JUND protein levels compared to the controls (*P* < 0.012 and *P* < 0.001, resp.) and no effect was exerted by mouse IgG (Figure 5). In addition, no effect was evidenced in FRA1 and FRA2 protein expression levels (data not shown).

**Figure 6:**
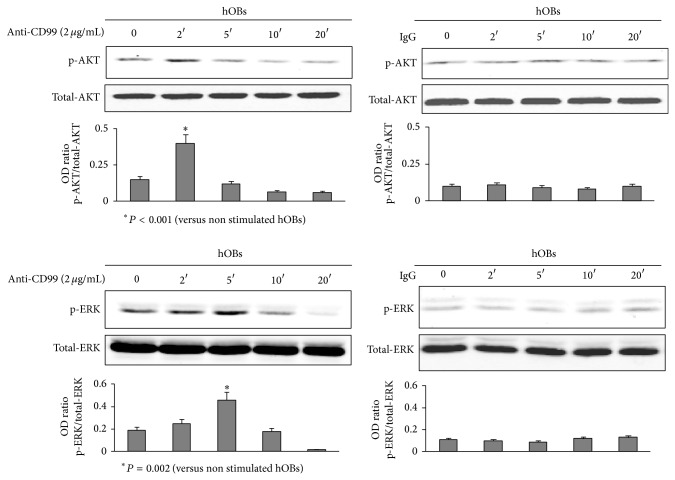
CD99 agonist monoclonal antibody induces AKT and ERK phosphorylation in normal human osteoblasts (hOBs). hOBs, treated with anti-CD99 agonist monoclonal antibody or with mouse IgG for 0, 2, 5, 10, and 20 minutes, were lysed and analyzed by western blot analysis to detect the protein levels of phosphorylated AKT (p-AKT, upper panel) and ERK (p-ERK, lower panel). The histograms represent the mean optical density (OD) of p-AKT or p-ERK ratio normalized to the OD of total-AKT or total-ERK, respectively. Data are presented as mean ± SE. The figure shows one representative of three independent experiments.

**Figure 7:**
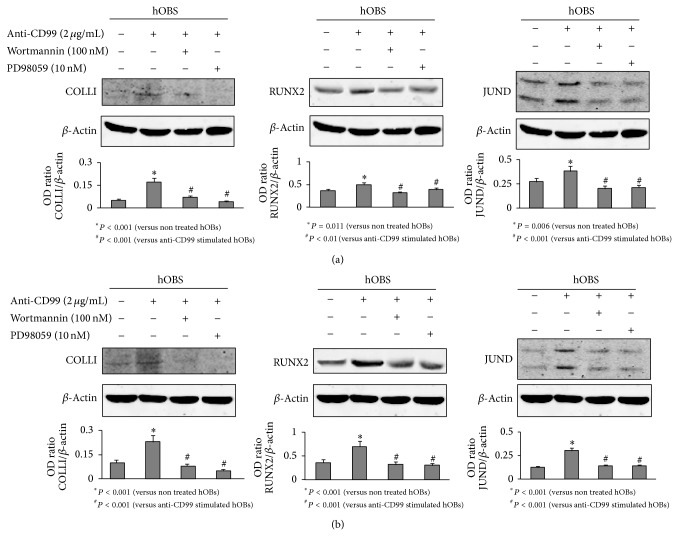
PI3K and ERK1 K inhibitors rescue Collagen I (COLLI), RUNX2, and JUND upregulation induced by CD99 agonist monoclonal antibody in normal human osteoblasts (hOBs). hOBs pretreated for 30 or 60 minutes with PI3K (Wortmannin) or ERK1 K (PD 98059) inhibitors, respectively, were stimulated for 4 (a) or 6 hours (h) (b) with anti-CD99 agonist monoclonal antibody and then lysed and analyzed by western blotting to detect the protein levels of COLLI, RUNX2, and JUND. The histograms represent the mean optical density (OD) of COLLI, RUNX2, or JUND ratio normalized to the OD of *β*-Actin. Data are presented as mean ± SE. The figure shows one representative of three independent experiments.

To identify the intracellular signal transduction molecules involved in CD99 signaling pathway(s) in our hOB culture system, the expression of several signaling mediators was examined after CD99 activation. In particular, we demonstrated that the anti-CD99 agonist antibody significantly induces the phosphorylation of signaling components such as AKT and ERK1/2 (*P* < 0.001 and *P* = 0.002, resp.). As shown in Figure 6, the CD99 activation induces AKT phosphorylation after 2 minutes of treatment and ERK1/2 phosphorylation after 5 minutes of stimulation, whereas the usage of mouse IgG did not induce any effect. JNK and P38 were not phosphorylated after CD99 activation (data not shown). To evaluate if AKT and ERK phosphorylation was responsible for COLLI, RUNX2, and JUND modulation mediated by CD99 activation, we studied the expression of these molecules in the presence of PI3K and ERK1 K inhibitors. We demonstrated that after 4 and 6 hours (Figures 7(a) and 7(b)) of CD99 stimulation, both PI3K (involved in AKT phosphorylation) [15] and ERK1 K inhibitors significantly rescue COLLI (*P* < 0.001), RUNX2 (*P* ≤ 0.01), and JUND (*P* < 0.001) protein levels in hOBs.

**Figure 8:**
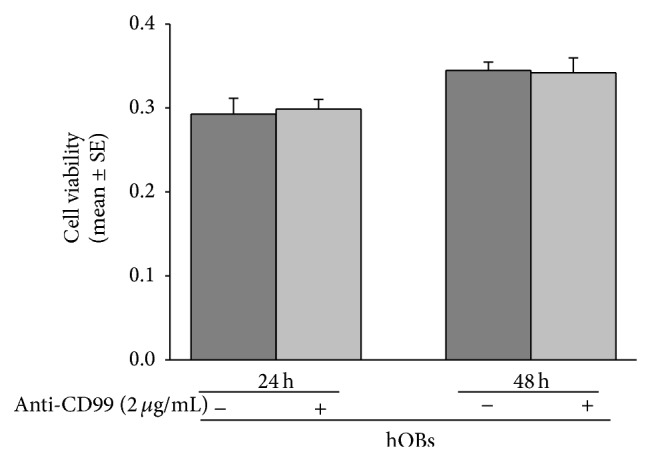
CD99 agonist monoclonal antibody does not induce the reduction of normal human osteoblast (hOBs) viability. Cell viability, evaluated by MTT assay, in hOBs treated for 24 or 48 h with anti-CD99 agonist monoclonal antibody. Results are expressed as values of optical density at 570 nm ± SE of three independent experiments performed in triplicate.

Finally, due to the knowledge that the activation of CD99 causes T-lymphocyte and thymocyte apoptosis [30, 31], in parallel to the previously described experiments, we investigate hOB sensitivity to CD99 activation induced apoptosis by analyzing cell viability through MTT assay. In particular, hOBs cultured in 96-well tissue-culture plates were treated for 24 and 48 hours with 2 *μ*g/mL of anti-CD99 agonist antibody. As shown in Figure 8, we found that the activation of CD99 failed to exert any effect on cell viability. To support this finding, we further studied the expression of signaling molecules involved in the apoptosis caspase-cascade events, such as caspase-8 (the initial caspase) and caspase-3 or caspase-7 (the effector caspases) [42, 43] as well as Bid, a death agonist member of the Bcl2/Bcl-xL family [44]. By western blot analysis, we demonstrated that hOBs treated for 4, 6, 12, and 24 hours with 2 *μ*g/mL of anti-CD99 agonist antibody do cause neither caspases 8, 3, and 7 fragmentation nor Bid cleavage (data not shown). In these experiments TRAIL stimulation, known to induce apoptosis in hOBs [45], was used as positive control (data not shown).

## 4. Discussion

In the present study we demonstrated that HMCLs or their conditioned media downregulate the expression of CD99 by hOBs during their differentiation process. Intriguingly, influenced by HMCLs, undifferentiated and differentiated hOBs, in addition to the reduced levels of CD99, display a less differentiated status. We further showed that CD99 is a critical molecule in the regulation of the physiological process of hOB differentiation and activity since the expression of ALP, COLLI, RUNX2 and JUND are upregulated by the activation of CD99 in hOBs. These findings suggested that the downregulated levels of CD99 could have a critical role in the well-known impairment of osteoblastogenesis and bone formation occurring in the osteolysis associated with MM.

Although CD99 is largely expressed in normal tissues [5] and recently linked to hOB differentiation, variable levels have been demonstrated in different pathological conditions. With particular regard to bone malignancies, a strong expression has been shown in Ewing's sarcoma whereas low levels have been detected in osteosarcoma. It has been shown that CD99-forced expression considerably affects osteosarcoma cell behavior reversing their cell malignancy by regulating critical biological processes required for metastases [5]. New data also provide evidence that when CD99 is restored in osteosarcoma cells, the molecule favors terminally differentiated phenotype [8].

It is worth noting that the supernatants from different tumors associated with osteolytic lesions or osteosclerotic metastasis can vary CD99 expression in osteoblastic cells [18]. However, no data are at present available in the literature regarding the possible modulation of CD99 expression in hOBs exerted by cells of MM, a neoplasm associated with osteolytic bone disease which is due not only to increased osteoclast activity but also to alteration of OB differentiation and function.

Increasing evidence demonstrate that MM cells can impair OB formation and activity through different cellular mechanisms including both secretion of soluble factors [25–29] and cellular contact, such as the interaction with stromal or OB cells [24, 27]. Therefore, based on the new findings demonstrating that differentiated OBs express high CD99 levels we first studied whether myeloma cells could influence the expression of this molecule and next if CD99 could be critical in the differentiation of hOBs.

In this work we found that HMCLs, H929 and U266, which weakly express CD99, induce a significant inhibition of CD99 expression by normal hOBs during their differentiation process. We also demonstrated that this inhibition, although significant, is less evident in the presence of the conditioned medium of both HMCLs indicating that the cell contact and, in a less way, the possible release of soluble molecules in the media induce CD99 reduction in hOBs.

On the basis of recent data demonstrating a high expression of CD99 in differentiated hOBs, we hypothesized that HMCLs through the reduction of CD99 could contribute to OB impairment.

Indeed, these latter cells cocultured with the two HMCLs express less RUNX2 and COLLI amount in favor of the hypothesis that the modulation of CD99 by myeloma cells, in addition to other mechanism(s), could take part in the induction of a less differentiated OB phenotype. In addition, a further support for this hypothesis comes from a very recent paper showing that CD99 expression in extramedullary biopsies of MM patients correlates with longer overall survival suggesting CD99 a new marker for risk stratification of disease severity [19]. These findings let us the possibility to consider the correlation between this* in vivo* study with our* in vitro* data in which the downregulation of CD99 could be involved in the alteration of osteoblastic differentiation and activity taking part in the onset of osteolysis in MM.

To prove that the reduction of CD99 we found could be directly responsible for the less differentiated status of OBs, it would be successful the use of a specific neutralizing antibody. However, a CD99 neutralizing antibody neither is at present available commercially nor is produced by some investigators. Thus, to overcome the difficulty of proving direct evidence that the CD99 reduction could take part in the impairment of osteoblastogenesis and bone formation occurring in the osteolytic process associated with MM, we investigated whether the activation of CD99 is able to modulate the activity of hOBs. At this purpose we performed different experiments on hOBs in the presence of a specific agonist antibody, widely used by other authors to achieve information on CD99 activities [32, 38, 39].

We here demonstrated that in hOBs the activation of CD99 stimulates the activity of ALP, the most widely recognized biochemical marker for hOB, and doubled the levels of COLLI, the most abundant organic component of bone matrix [46–48]. Such data point out that, by forcing the function of CD99, hOBs result more active in the expression of their differentiation parameters. This is in agreement with our previous demonstration showing that whenever CD99 expression was regained by osteosarcoma cells, they reactivate the terminal osteoblastic differentiation program [8]. Now, we also provide evidence that in hOBs CD99 activation contributes to increase the protein levels of RUNX2, the master transcription factor for OB differentiation which is central in triggering the expression of major bone matrix protein genes including the COLLI [49]. In parallel, we further demonstrated high levels of JUND, member of AP1 heterodimeric complex, crucial regulator of osteogenic genes that acts as coregulator of RUNX2 itself [50, 51]. We further found that in hOBs CD99 activation induces AKT and ERK phosphorylation, consistent with data demonstrating the ability of CD99 stimulation to induce MAPKs and protein kinase C activation [52, 53] and findings showing that, in human breast cancer cells, CD99 promotes SRC, AKT, ERK, and JNK activation, thus increasing JUND and FOSB AP-1 transcription factors expression [15]. In line with the recent demonstration that CD99-restored expression in osteosarcoma cell correlated with ERK 1/2, RUNX2, and AP-1 activation [8], we here show that PI3K and ERK1 K inhibitors rescue COLLI, RUNX2 and JUND OB protein levels. Therefore, we can here assess that CD99 activation, through the phosphorylation of AKT and ERK 1/2, increases RUNX2 and JUND transcription factors as well as COLLI levels, thereby playing a significant role in the activation of normal hOBs.

## 5. Conclusions

Our results highlight an important role of CD99 in the differentiation and activity of hOBs in physiological and pathological conditions. HMCLs induce a reduction of CD99 expression in hOBs which display a less differentiated phenotype, suggesting a possible contribution of this molecule in the impairment of osteoblastogenesis occurring in MM bone disease. Indeed, by forcing the function of CD99 the hOBs result more active in the expression of their differentiation parameters.
